# Electrophysiological Characterization of Cerebellar Responses during Exploration and Grooming Behaviors in a Rat Model of Parkinsonism

**DOI:** 10.3390/brainsci13040537

**Published:** 2023-03-24

**Authors:** Lizbeth Vásquez-Celaya, Gerardo Marín-Márquez, Jorge Manzo, Porfirio Carrillo-Castilla, Armando Jesús Martínez, Ricardo Ortiz Pulido, René Zempoalteca Ramírez, Genaro A. Coria-Avila, Luis I. García

**Affiliations:** 1 Instituto de Investigaciones Cerebrales, Universidad Veracruzana, Xalapa 91190, Mexico; 2 Neural Dynamics and Modulation Lab, Cleveland Clinic, Cleveland, OH 44195, USA; 3 Instituto de Neuroetología, Universidad Veracruzana, Xalapa 91190, Mexico; 4 Dirección de Actividades Deportivas, Universidad Veracruzana, Veracruz City 91020, Mexico; 5 Centro Tlaxcala de Biología de la Conducta, Universidad Autónoma de Tlaxcala, Tlaxcala 90070, Mexico

**Keywords:** Parkinsonism, basal ganglia, cerebellum, multiunit activity, electrolytic lesion, ventrolateral striatum

## Abstract

Parkinson’s disease is currently a global public health challenge due to the rapid growth of aging populations. To understand its pathophysiology is necessary to study the functional correlation between the basal ganglia (BG) and the cerebellum, which are involved in motor control. Herein, we explored multiunit electrical activity (MUA) in the cerebellum of rats with induced Parkinsonism as a result of lesions following bilateral placement of electrodes and passing of current in the ventrolateral striatum (VLS). In one control group, the electrodes descended without electrical current, and another group was left intact in VLS. MUA was recorded in Sim B and Crus II lobes, and in the dentate nucleus (DN) during the execution of exploration behaviors (horizontal and vertical) and grooming. The lesioned and sham groups showed a decrease in MUA amplitude in the Crus II lobe compared to the intact group in all recorded behaviors. However, Sim B and DN did not express differences. Both electrical and physical insults to the VLS induced Parkinsonism, which results in less MUA in Crus II during the execution of motor behaviors. Thus, this type of Parkinsonism is associated with a decrease in the amplitude of Crus II.

## 1. Introduction

Parkinson’s disease (PD) is a chronic neurodegenerative pathology of the nervous system with an incidence of 159% worldwide in the last 30 years, which makes it a public health challenge mainly due to the rapid growth of aging populations [1]. The origin of PD is still unknown; however, the progressive loss of dopaminergic neurons of the substantia nigra pars compacta (SNc) results in decreased dopamine (DA) to the striatum (caudate/putamen), mainly affecting the cortico-basal motor circuit [2,3,4]. However, motor control also depends on the integrity of other connections, such as those between the basal ganglia (BG) and the cerebellum, which are both anatomical [5,6,7] and functional [8,9,10,11].

Indeed, alterations in the BG may result in Parkinsonism, which is a characteristic syndrome of PD observed as tremors at rest, joint stiffness, bradykinesia-hypokinesia and postural alteration [3,12]. For instance, a type of Parkinsonism (i.e., tremulous jaw movements, TJMs) may be caused by subchronic systemic treatment with the dopamine antagonist haloperidol. Rats with haloperidol-induced TJMs express less cerebellar activity in the vermis (as observed via cFOS immunoreactivity), but more in the hemispheres, particularly in the dorsal granular region of lobes Simple B (Sim B), anxiform lobe 2 (Crus II), and in the deep Dentate Nucleus (DN) [11]. Interestingly, TJMs can also be experimentally induced when the integrity of the ventrolateral striatum (VLS) is compromised [13,14]. Thus, to contribute to the pathophysiology of Parkinsonism, the present study explored the cerebellar electrophysiological response via multiunit activity (MUA) in rats with TJMs induced by a lesion in the VLS [11]. The animals were randomly divided into three groups: intact, sham and lesion MUA was recorded individually in Sim B, Crus II, and DN during the execution of the exploration behavior (horizontal and vertical) and grooming.

## 2. Materials and Methods

### 2.1. Subjects and Housing

Thirty-six Wistar male rats (250 to 350 g) were housed in acrylic boxes (44 × 33 × 20 cm), with wooden chip bedding (Rismart, México City, Mexico), in a room with an inverted light-dark cycle for periods of 12 × 12 h and maintained with rodent feed (Purina Rodent Chow^®^, México City, Mexico) and drinking water ad libitum. The experimental protocol followed the Guide for Care and Use of Laboratory Animals (NOM/GCUAL) and the animal use policies stipulated by the Society of Neuroscience and the Official Mexican Norm (NOM-062-ZOO-1999). All the animals were housed in the vivarium of the Brain Research Institute at Universidad Veracruzana.

### 2.2. Study Groups

Rats were randomly organized into three groups. The lesioned group had an electrode lowered bilaterally in the VLS and received an electrical current to induce the electrolytic lesion; in the sham group, the electrodes were lowered but with no electrical current, and in the third group, rats were left intact.

### 2.3. Grooming

Grooming behavior was analyzed because it is part of the normal repertoire of rats. It facilitates the maintenance of hygiene and physiological processes such as thermoregulation, social communication, and de-arousal. Grooming was considered as such when rats displayed self-licking of flanks, abdomen, genitals, and tail or face-washing using their forelimbs around the muzzle and tongue [15].

### 2.4. Exploratory Behavior

Exploration behavior requires locomotion so that animals gain information about their environment [16]. Exploratory behavior or locomotion in the laboratory rat is evaluated in a variety of test situations. Horizontal exploration was considered when the rat wandered inside the acrylic box in the horizontal plane. Vertical exploration was considered when the rat was upright and performed exploratory movements with its head toward the environment [17].

### 2.5. Electrode Implant and Recording

Before stereotaxic surgery, the rats were deeply anesthetized with an intraperitoneal injection of Ketamine (100 mg/kg, Pisa, México City, Mexico) and Xylazine (8 mg/Kg, Cheminova, México City, Mexico). Anesthesia was confirmed by checking the absence of the flexor reflex in the lower limbs. Then, rats were shaved and cleaned in the dorsal area of the skull and placed in a stereotaxic device (Stoelting Co. 620, Wood Dale, IL, USA). A longitudinal incision was made in the dorsal region of the head of approximately 2 cm. The area was cleaned by removing the aponeurosis until the cranial sutures, bregma, and lambda were exposed. Local anesthesia and vasoconstrictors were applied (Adrecaine Aranda Lab^®^, México City, Mexico) on the wound to reduce sensitivity and prevent bleeding, respectively. A trepanation (Dremel Rotatory Tools^®^, Racine, WI, USA) was carefully performed on the skull in order to place the monopolar stainless-steel electrodes (FHC, Inc., Bowdoin, ME, USA 250 μm θ, 3 MΩ) on each structure. The coordinates used for the VLS corresponded to anteroposterior (AP) = −4.0 mm, mediolateral (ML) = ±4.4 mm, and dorsoventral (DV) = −6.6 mm; in the lobe Sim B the coordinates were: AP = −12.4 mm, ML = 3.4 mm and DV = −2.8 mm; for Crus II lobe: AP = −14 mm, ML = 3.40 mm, and DV = −5 mm and for the ND: AP = −11.3 mm, ML = 3.40 mm, and DV = −6.4 mm. At the same time, a stainless-steel screw (Stoelting, Co., Wood Dale, IL, USA) was implanted in the skull as a reference electrode [18].

After 48 h of recovery from stereotaxic surgery, MUA records were carried out in the granular layer of Sim B and Crus II lobes, as well as in DN (n = 4 for each subgroup), one every 24 h. Before MUA recording, the rats were left undisturbed for five minutes in an acrylic box (30 × 30 × 30). Then, the recording and reference electrodes were connected to an interface that transferred the analog signal to amplifiers 15A54, 15LT (Grass Technologies, Inc. West Warwick, RI, USA), and an audio monitor (AM9, Grass Technologies^®^, Inc., West Warwick, RI, USA). The amplified signal was sent to the PVA-16 Polyview Adapter System (Grass Technologies^®^, Inc., West Warwick, RI, USA) to be digitized and then sent to a computer (HP 6730b, Palo Alto, CA, USA), where the traces of each animal were stored in a recording program (Polyview Recording System, Grass Technologies^®^, Inc., West Warwick, RI, USA). During the recordings, marks were placed on the MUA traces, indicating the observed behavior while simultaneously videotaping the animals.

### 2.6. Electrolytic Lesion and Tissue Processing

Using a stimulator (Stimulator S48 Grass Astro-Med Inc., West Warwick, RI, USA), a Stimulus Isolation Unit SIU, and the Constant Current Unit CCU1, 3.5 mA direct current was applied for 30 s through an electrode placed on the VLS. Subsequently, trepanation was performed on the skull corresponding to the area to be recorded. A recording electrode was lowered and fixed in the cerebellum according to the coordinates indicated in the stereotaxic atlas.

After the surgery, dental acrylic was placed to fix the electrodes (record and reference), and the animals were placed in a recovery chamber to prevent them from falling into hypothermia. Once recovered, they received post-surgical treatment with systemic analgesic and antibiotic (Flunixin meglumine 2.5 mg/kg and enrofloxacin 5 mg/kg) and returned to the vivarium.

After electrophysiological recordings, transcardiac perfusion was performed; Thus, animals were sacrificed with intraperitoneal sodium pentobarbital (160 mg/kg, Pisa, México City, Mexico). An incision was made below the sternum and continued at both ends of the ribs, perforating the diaphragm to leave the heart exposed; with a clamp, the flow of the abdominal descending aorta was stopped, and through a cannula inserted in the left ventricle of the rat, 0.9% saline (300 mL) and 4% paraformaldehyde solution (300 mL) were administered systemically with a constant flow of approximately 23.4 mL per minute. Following perfusion, the cerebellum was extracted to be stored in paraformaldehyde for 24 h, then in 10%, 20%, and 30% glucose for 72 h. Coronal and sagittal slices of the brain and cerebellum with a thickness of 50 microns were obtained in a cryostat (Leica^®^ CM1850, Nussloch, Germany) at −24 °C, then the tissue was observed under a light microscope (AX70 Olympus Co., Shinjuku, Tokyo, Japan) using 10× and 40× objectives to corroborate that the electrode was implanted in the correct structure.

### 2.7. Statistical Analysis

Ten three-second traces were selected during grooming, horizontal, and vertical exploration. Then, we compared the MUA of three brain regions for each behavior. The response variable was analyzed with a Generalized Linear Model (GLM) in a fixed and nested (hierarchical) factor design in pseudorepetition analysis [10,19,20]:

(1) $$y=G+S+G*S+R_{\left[S\right]}+T_{\left[R\right]}+error$$

where *y* is the response variable (amplitude of the recorded behavior) for each behavior analyzed (grooming, horizontal exploration, and vertical exploration) that corresponded to a separate analysis. This is because the traces of multiunit activity are hierarchical (pseudorepetition) in the Sim B and Crus II lobes and the dentate nucleus, it is justified to adjust the hierarchical GLM and control the effect of pseudorepetition to avoid type II statistical errors [10,21].

Where *G* is the group with three levels (intact, sham, and lesion), *S* is the recording structures (Sim B, Crus II, and DN), *G × S* is the interaction between the groups and the structures, R_[*S*]_ represents the rats in which each structure was nested and traces T_[*R*]_ which are the pseudorepetition of each of the rats (ten traces (T) per record of each behavior of each rat and nesting is indicated in square brackets in the statistical model). All analyses were performed in statistical packages (JMP Pro 14.0.0, SAS 2018 Institute Inc., Cary, NC, USA) where a post hoc Tukey test was performed to determine differences between structures and groups and delimited by the effect of the interaction registered in the statistical model. The assumptions of normal error distribution and homogeneity of variances were verified. Graphics software development was used (SigmaPlot 10, Systat^®^ Software Inc., San Jose, CA, USA). The data are shown as the mean ± standard error (SEM). All the traces were randomly selected for each structure and each group.

## 3. Results

Recordings of animals that were not adequately implanted were discarded from the study. In general, we found decreased MUA amplitude in the Crus II lobe of both lesioned and sham groups compared to the intact group in all recorded behaviors. In Sim B and the DN, we did not observe differences.

### 3.1. Grooming

**Figure 1:**
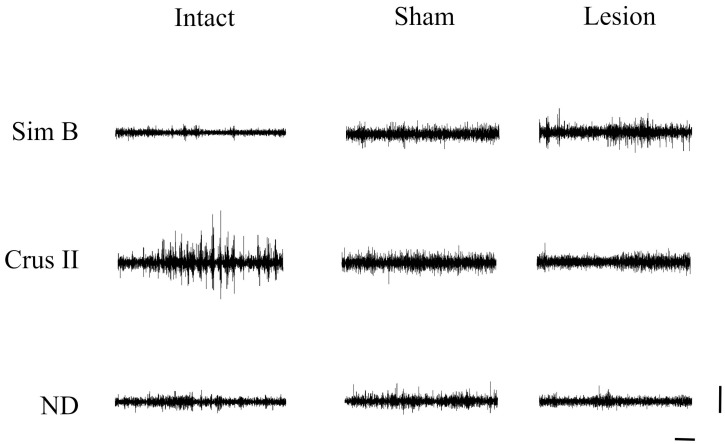
Representative traces of multiunit activity of three seconds were obtained during the grooming behavior of animals from intact, sham, and lesion groups in the Sim B and Crus II lobes and the dentate nucleus. Horizontal calibration: 500 ms. Vertical Calibration: 2 mV.

**Figure 2:**
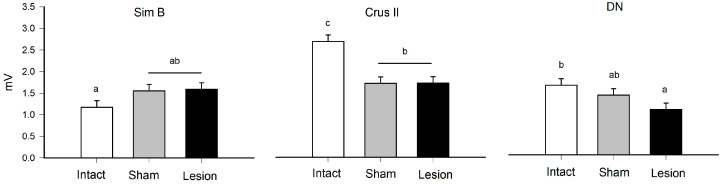
Mean value (mean ± SE) of the maximum amplitude (mV) during grooming behavior in Sim B, Crus II, and the dentate nucleus (DN) in the intact, sham, and lesion groups. The different letters on the bars (a, b, and c, *α* = 0.05) indicate contrast in multiple comparisons by Tukey’s method for the means recorded in the groups of rats and structure that were delimited by the effect of the interaction corresponding to the statistical model (Table 1).

Both sham and lesioned groups showed decreased MUA in the Crus II lobe amplitude. This may indicate this structure is sensitive to modifications of the thalamus-cerebellar connections (Figure 1 and Figure 2) (Table 1). In the effects test, we found that the interaction between group and structure presented significant differences (Table 1), with *p* < 0.0001, observing modifications of the amplitude recorded for the groups that received alteration in the VLS.

### 3.2. Horizontal Exploration

**Figure 3:**
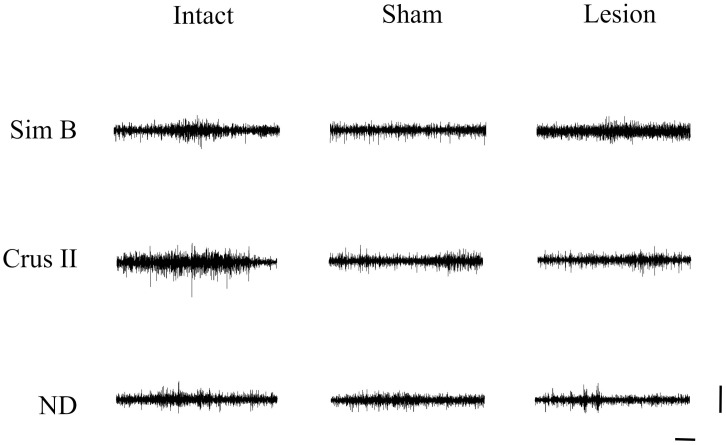
Representative traces of three-second multiunit activity were obtained during horizontal exploration of animals from intact, sham, and lesion groups in the Sim B, Crus II lobes, and the dentate nucleus. Horizontal calibration: 500 ms. Vertical Calibration: 2 mV.

**Figure 4:**
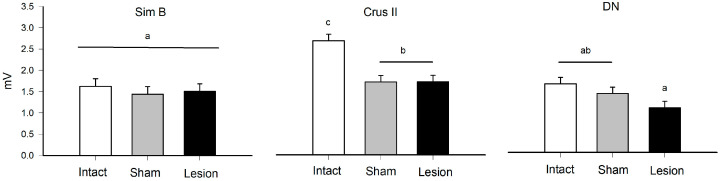
Mean value (mean ± SE) of the maximum amplitude (mV) during horizontal exploration of the Sim B, Crus II structures, and the dentate nucleus (DN) in the intact, sham, and lesion groups. The different letters on the bars (a, b, and c, *α* = 0.05) indicate contrast in multiple comparisons by Tukey’s method for the means recorded in the groups of rats and structure that were delimited by the effect of the interaction corresponding to the statistical model (Table 2).

When comparing the MUA between the groups, we found that the animals that received a lesion in the VLS presented a decreased MUA in the Crus II lobe compared to the intact group. No significant differences were observed for the Sim B lobe and dentate nucleus (Figure 3 and Figure 4) (Table 2).

### 3.3. Vertical Exploration

**Figure 5:**
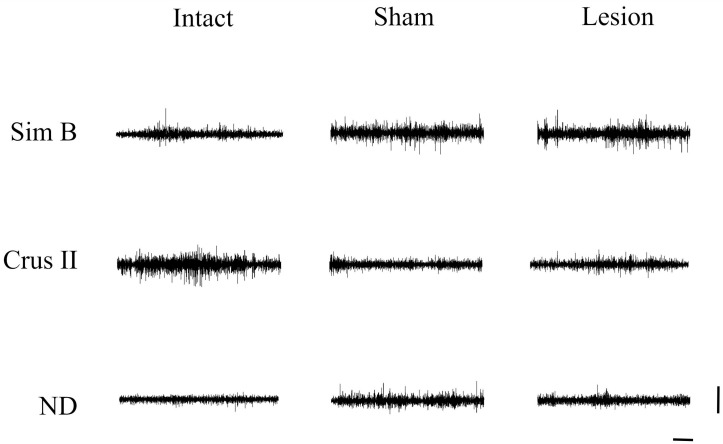
Representative traces of three-second multiunit activity were obtained during vertical exploration of animals from intact, sham, and lesion groups in the Sim B, Crus II lobes, and dentate nucleus. Horizontal calibration: 500 ms. Vertical Calibration: 2 mV.

**Figure 6:**
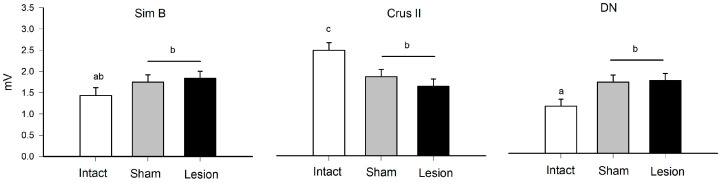
Average value (mean ± SE) of the maximum amplitude (mV) during vertical exploration of the Sim B, Crus II structures, and the dentate nucleus in the intact, sham, and lesion groups. The bifferent letters on the bars (a, b, and c, *α* = 0.05) contrast in multiple comparisons by Tukey’s method for the means recorded in the groups of rats and structure that were delimited by the effect of the interaction corresponding to the statistical model (Table 3).

MUA was decreased in the Crus II lobe of sham and lesioned groups (Figure 5 and Figure 6). No differences were observed for the other groups. The effects test showed significant differences when comparing the interaction between group and structure (Table 3).

## 4. Discussion

### 4.1. Summary and Contributions

The present study was designed to explore the physiological interrelationship between the ventrolateral striatum (VLS) and the cerebellum. We analyzed multiunit activity (MUA) in the hemispheric nuclei Sim B, Crus II, and in the deep dentate nucleus of rats with induced Parkinsonism after lesioning the VLS.

Our results showed that of those structures, only Crus II expressed decreased MUA during grooming, horizontal, and vertical exploration behavior in both sham and lesioned groups. Accordingly, the decrease in MUA is related to both the electrolytic lesion (as in the lesioned group) and the mechanical insult (as in the sham group). Both types of insults in the VLS may have caused modifications in the functional relationship of the VLS with Crus II via the striatum internal globus pallidus (Gpi) subthalamic nucleus (STN) thalamus cerebellar pathway. Of these structures, the STN projects to the pontine nuclei, which are the source of excitatory mossy fibers to the granular layer of the cerebellar cortex (including Crus II) and the deep nuclei [22,23]. Thus, the decrease in MUA in Crus II may correspond to disinhibition from VLS. According to a putative direct pathway, a lesioned VLS would be incapable of inhibiting the GPi and SNr. Active GPi and SNr would inhibit the reticular formation (RF) and superior culliculus (SC), which in turn, would release the dorsolateral nucleus of the trigeminal nerve (DLNT) to cause TJMs [24].

On the other hand, there may be an indirect pathway. A lesioned VLS would not inhibit external globus pallidus (GPe). Disinhibition of GPe would increase its inhibitory effect on the STN, which would not excite the GPi, resulting in the abnormal activity of RF and DLNT. Both pathways, direct and indirect, would result in TJMs [10], which would evoke alterations in the cerebellum. For instance, a former study from our group showed that during TJMs, the cerebellum responded with more (compensatory) activity, as observed via greater expression of c-Fos in the lobes Sim B, Crus II, and dentate nucleus [25].

Interestingly, TJM was expressed in males receiving an electrolytic lesion but also in those from the sham group with electrodes placed (without electrical current) in the VLS. Accordingly, an electrical current passing through the VLS was not needed to produce TJMs, but only descending the electrodes [10]. The results of the present study confirm such an effect and show that insults to the VLS that cause TJMs are associated with decreased baseline amplitude in Crus II.

A smaller spike amplitude in Crus II may reflect less active cells, which would not allow an effective function. However, the amplitude reduction did not correspond to inadequate motor performance in those animals from lesion or sham groups. Thus, the increased expression of c-Fos [25] and the decreased baseline MUA of this structure [11] may not only correspond to motor activity but perhaps to other functions that the cortex of this lobe fulfills. All animals were capable of performing the evaluated behaviors, regardless of the group to which they belonged. It is relevant because it allows us to correlate the observed MUA with the role played by the structures recorded in each behavior without considering the effect that any deficiency in the peripheral motor skills of the animals may imply.

Returning to the paradigm that a greater amplitude of the MUA implies neuronal recruitment, a decrease may indicate a regulation of the cerebellar system to perform the evaluated tasks. Thus, to understand the function of the cerebellar system, it is necessary to know the critical role of the Purkinje cells as an efferent pathway from the cerebellar cortex to the deep nuclei cells of the cerebellum, and its projections to the rest of the nervous system, as well as climbing fibers, mossy fibers, granular cells and parallel fibers, which are indispensable to explain the cerebellar functional circuit. Thus, regarding the motor circuit, the information coming from the cerebral cortex is sent to the basal ganglia to determine adequate motor execution through the direct and indirect pathways [26,27]. There is a connection between the striatum, GPi, and GPe. The GPi projects to the thalamus and back to the cortex. In addition, the GPe has connections to the STN, which closes the circuit by stimulating the GPi. There are direct projections to the cerebellar cortex, considering that the alteration caused in the striatum mainly affects the indirect pathway causing TJMs. The lack of inhibition of the indirect pathway is due to the neuronal loss caused by a lesion on the VLS [28]. In addition, we cannot rule out the presence of neuroinflammation due to the electrolytic lesion. Such insult to the circuit may generate “erroneous” information about what is happening in the motor pathways, altering other areas involved in the motor response, including the cerebellum [29].

In the case of Sim B, we found no differences between the groups for any of the behaviors recorded, which may suggest a process of internal cerebellar regulation that compensates for the functions altered by the lesion in the VLS, allowing the animals to perform an efficient grooming and exploration execution, altered only by the presence of mandibular tremor. This functional possibility could also be applied to DN as an area that processes the sum of excitatory and inhibitory potentials from the cerebellar cortex and the cerebellar afferent pathways. That is, the cerebellar cortex system plays the role of an internal system of self-regulation.

Evidence indicates that the cerebellum receives kinesthetic information from the extremities through the spinocerebellar tracts and the face and head through the cerebellar trigeminal tract. In addition to afferent information on retransmission of the lower olivary complex through the spine-olivary tract [30]. On the one hand, the main olive core sends climbing fibers to lobe VII and DN [31], while the cortex of the parietal lobe is interconnected reciprocally with lobe VII and cerebellar lobes Crus I and Crus II. On the other hand, [31,32], there is evidence that language-related activity is focused on the lateral and posterior regions of the cerebellum, including lobes VI, Crus I, and II. Working memory paradigms also activate posterior VI lobes/Crus I areas. Identifying emotional intonation produces cerebellar activation in lobe VII and the lateral posterior hemisphere (lobe VI bilaterally and right Crus I) [32]. The above could indicate that the functional role of the lobes Crus I and Crus II implies much more than a single role in the cerebellum. The fact that these structures are related to processes of working memory, motor response, and language could mean that the differences we find are due to the final computation of the integration of each of them. We hold in mind, however, that MUA does not discriminate between neuronal types because it is a record of a wide area. Further research is needed to unravel the critical role of the cerebellum in functions that were for a long time merited only to the brain, such as those that result in the motor alterations observed in Parkinson’s disease.

### 4.2. Strength and Limitations

Basic science studies such as ours have set the pattern of association and compensation that the cerebellum can suffer from parkinsonism-type motor disorders; however, the functional correlation between the cerebellar lobes of rats and humans varies, despite this, there are already studies who are investigating the role of the cerebellum in patients with Parkinson’s disease [33,34], where an increase in cerebellar activity of lobules VI and crus II was observed in motor and cognitive walking tests.

The findings of the present study relate cerebellar function with motor activities that are usually affected in parkinsonism models. The relationship between the cerebellum and motor alterations in patients with Parkinson’s has already begun to be studied. However, our project cannot be transposed directly to humans due to the complexity of the cerebellar functions that have been described. However, basic science studies are the foundations that point to the cerebellum as a target organ to study to understand the motor alterations of patients and its possible stimulation that can promote compensation with a view to lessening the motor alterations of the patients.

### 4.3. Future Work

Further research is needed to unravel the critical role of the cerebellum in functions that were, for a long time merited only to the brain, such as those that result in motor alterations observed in Parkinson’s disease. For that, we suggested carrying out behavioral tests that evaluate if there is a difference between the groups analyzed in other behaviors, such as the frequency of tremor appearance, as well as check if there is a modification in other cerebellar regions, such as the vermis. Real-time functional imaging tests could indicate other areas involved in the cerebellar response to locomotor execution and could clarify whether the modification of the cerebellar AMU is due to compensation or alteration of the mechanical lesion.

## 5. Conclusions

The present study showed that as a result of VLS-induced Parkinsonism, the cerebellum responded with less MUA in specific regions, such as Crus II, during the execution of motor behaviors. This suggests that Parkinsonism is associated with a decrease in the amplitude of Crus II. Future research will determine if the modifications of the cerebellar MUA suppose a functional response that covers the deficiencies in the traditional motor circuits.

## Figures and Tables

**Table 1:** Effects test for grooming behavior. Results of nested ANOVA for the effect of traces (T), rat (R), structure (S), group (G), and probability of differences based on F values and *r*^2^ = 0.37 of the model. The animals used were made in independent groups.

Source	DF	Sum of Squares	Ratio F	*p*	Post Hoc
Group	2	9.00	4.93	0.001	I > S, L
Structure	2	31.93	17.49	<0.00001	C > Si, D
Group × Structure	4	26.78	7.33	<0.0001	see Figure 2
Rat [Structure]	9	83.07	10.11	<0.0001	
Traces [Rat]	36	13.32	0.40		NS

NS = not significant, I = Intact, S = Sham, L = Lesion, Si = Sim B, C = Crus II, D = DN. The multiple comparisons of the interaction Group × Structure are shown in Figure 2 and at the nesting level it was not necessary to perform the post hoc comparisons.

**Table 2:** Effects test for horizontal exploration behavior. Results of nested ANOVA for the effect of traces (T), rat (R), structure (S), group (G), and probability of differences based on F values and *r*^2^ = 0.33 of the model. The animals used were made in independent groups.

Source	DF	Sum of Squares	Ratio F	*p*	Post Hoc
Group	2	22.15	8.50	<0.0001	I > S, L
Structure	2	17.42	6.69	<0.001	C, Si > D
Group × Structure	4	26.98	5.17	<0.0001	see Figure 4
Rat [Structure]	9	104.59	8.92	<0.0001	
Traces [Rat]	36	29.01	0.61		NS

NS = not significant, I = Intact, S = Sham, L = Lesion, Si = Sim B, C = Crus II, D = DN. The multiple comparisons of the interaction Group × Structure are shown in the Figure 4 and at the nesting level it was not necessary to perform the post hoc comparisons.

**Table 3:** Effects test for vertical exploration behavior. Results of nested ANOVA for the effect of traces (T), rat (R), structure (S), group (G), and probability of differences based on F values and *r*^2^ = 0.32 of the model. The animals used were made in independent groups.

Source	DF	Sum of Squares	Ratio F	*p*	Post hoc
Group	2	0.47	0.20	0.81	NS
Structure	2	13.26	5.79	<0.003	C > Si, D
Group × Structure	4	26.25	5.73	<0.0001	see Figure 6
Rat [Structure]	9	94.03	9.13	<0.0001	
Traces [Rat]	36	27.03	0.65		NS

NS = not significant, I = Intact, S = Sham, L = Lesion, Si = Sim B, C = Crus II, D = DN. The multiple comparisons of the interaction Group × Structure are shown in Figure 6 and at the nesting level it was not necessary to perform the post hoc comparisons.

## Data Availability

The data that support the findings of this study are available from the corresponding author.

## Abbreviations

µm	Micrometer
ANOVA	Nested analysis of variance
AP	Anteroposterior
BG	Basal ganglia
CCU1	Constant Current Unit
Cm	Centimeter
Crus II	Anxiform lobe 2
DA	Dopamine
DLNT	Dorsolateral nucleus of the trigeminal nerve
DN	Deep Dentate Nucleus
DV	Dorsoventral
GCUAL	Guide for Care and Use of Laboratory Animals
GLM	Generalized Linear Model
GPe	External globus pallidus
Gpi	Internal globus pallidus
kg	Kilograms
mA	Milliamps
mg	Milligrams
ML	Mediolateral
ml	Milliliters
ms	Milliseconds
MUA	Multiunit electrical activity
mV	Millivolts
MΩ	Milliohm
PD	Parkinson’s disease
RF	Reticular formation
SC	Superior culliculus
SE	Standard Error
Sim B	Lobes Simple B
SIU	Stimulus Isolation Unit
SNc	Substantia nigra pars compacta
STN	Subthalamic nucleus
TJMs	Tremulous jaw movements
VLS	Ventrolateral striatum
